# Poly[[μ_2_-2,2′-diethyl-1,1′-(butane-1,4-di­yl)diimidazole-κ^2^ *N* ^3^:*N* ^3′^](μ_2_-5-hydroxy­isophthalato-κ^2^ *O* ^1^:*O* ^3^)zinc]

**DOI:** 10.1107/S1600536811039377

**Published:** 2011-10-05

**Authors:** Ying-Ying Liu, Xing Wang, Yong-Sheng Yan

**Affiliations:** a Department of Chemistry and Chemical Engineering, Jiangsu University, Zhenjiang 212013, People’s Republic of China

## Abstract

In the title coordination polymer, [Zn(C_8_H_4_O_5_)(C_14_H_22_N_4_)]_*n*_, the Zn^II^ cation is coordinated by an O_2_N_2_ donor set in a distorted tetra­hedral geometry. The Zn^II^ ions are linked by μ_2_-OH-bdc (OH-H_2_bdc = 5-hy­droxy­isophthalic acid) and bbie ligands [bbie = 2,2′-diethyl-1,1′-(butane-1,4-di­yl)diimidazole], forming a two-dimensional layer parallel to the *ab* plane. The layers are further connected through intermolecular C—H⋯O and O—H⋯O hydrogen bonds, forming a three-dimensional supramolecular structure. In the bbie ligand, the two C atoms in the ethyl group are each disordered over two positions with a site-occupancy ratio of 0.69:0.31.

## Related literature

For background information on bis­(imidazole) ligands, see: Kan *et al.* (2011 ▶); Liu *et al.* (2007 ▶).
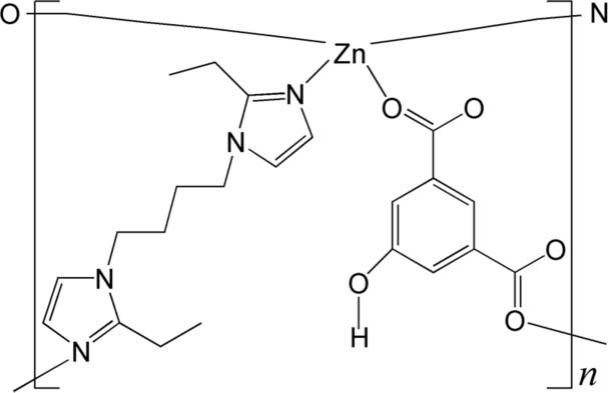


## Experimental

### 

#### Crystal data

[Zn(C_8_H_4_O_5_)(C_14_H_22_N_4_)]
*M* *_r_* = 491.84
Monoclinic, 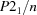

*a* = 13.5120 (12) Å
*b* = 12.765 (1) Å
*c* = 13.5910 (12) Å
β = 103.933 (2)°
*V* = 2275.2 (3) Å^3^
*Z* = 4
Mo *K*α radiation
μ = 1.12 mm^−1^
*T* = 293 K
0.28 × 0.24 × 0.21 mm

#### Data collection

Bruker SMART APEX CCD area-detector diffractometer
Absorption correction: multi-scan (*SADABS*; Sheldrick, 1996 ▶) *T*_min_ = 0.73, *T*_max_ = 0.79
13890 measured reflections
5467 independent reflections
3988 reflections with *I* > 2σ(*I*)
*R*_int_ = 0.034

#### Refinement

*R*[*F* ^2^ > 2σ(*F* ^2^)] = 0.036
*wR*(*F* ^2^) = 0.087
*S* = 1.03
5467 reflections
309 parameters
H-atom parameters constrained
Δρ_max_ = 0.32 e Å^−3^
Δρ_min_ = −0.29 e Å^−3^

### 

Data collection: *SMART* (Bruker, 2007 ▶); cell refinement: *SAINT* (Bruker, 2007 ▶); data reduction: *SAINT*; program(s) used to solve structure: *SHELXS97* (Sheldrick, 2008 ▶); program(s) used to refine structure: *SHELXL97* (Sheldrick, 2008 ▶); molecular graphics: *SHELXTL* (Sheldrick, 2008 ▶); software used to prepare material for publication: *SHELXTL*.

## Supplementary Material

Crystal structure: contains datablock(s) global, I. DOI: 10.1107/S1600536811039377/fj2452sup1.cif

Structure factors: contains datablock(s) I. DOI: 10.1107/S1600536811039377/fj2452Isup2.hkl

Additional supplementary materials:  crystallographic information; 3D view; checkCIF report

## Figures and Tables

**Table:** 

Zn1—O1	1.9662 (15)
Zn1—O4^i^	1.9682 (14)
Zn1—N4^ii^	2.0020 (18)
Zn1—N1	2.0286 (17)

**Table:** 

O1—Zn1—O4^i^	102.54 (6)
O1—Zn1—N4^ii^	117.20 (7)
O4^i^—Zn1—N4^ii^	118.18 (7)
O1—Zn1—N1	115.96 (7)
O4^i^—Zn1—N1	98.33 (6)
N4^ii^—Zn1—N1	103.59 (7)

Symmetry codes: (i) 
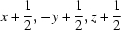
; (ii) 
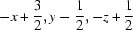
.

**Table 2:** Hydrogen-bond geometry (Å, °)

*D*—H⋯*A*	*D*—H	H⋯*A*	*D*⋯*A*	*D*—H⋯*A*
C10—H10⋯O1^iii^	0.93	2.62	3.417 (3)	144
C14—H14⋯O5^iii^	0.93	2.49	3.398 (3)	166
O5—H5⋯O2^iv^	0.82	1.88	2.697 (2)	171

Symmetry code: (iii) 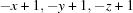; (iv) 
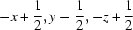
.

**Fig. 1.**
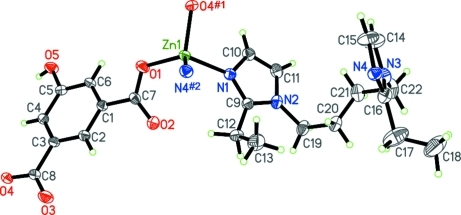
A view of the local coordination of the ZnII cation of (I). Displacement ellipsoids are drawn at the 30% probability level.

**Fig. 2.**
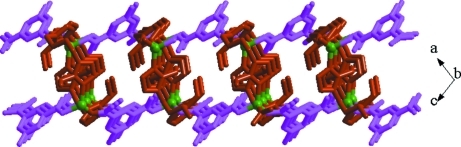
View of the two-dimensional layer of (I).

## supplementary crystallographic information

### Comment

As part of an investigation of the transition metal application there is a need to prepare further examples of these compounds. In this paper, the structure of the title compound, (I), is described.

As shown in Fig. 1, the Zn^II^ ions is four-coordinated by two oxygen atoms from two oxygen atoms of two OH-bdc anions and two nitrogen atoms of two bbie ligands. Each carboxylate group of OH-bdc acts in a monodentate mode, and the hydroxyl group is not involved in coordination. The bbie molecule coordinates to two Zn^II^ cations through its two aromatic N atoms, thus acing as a bridging bidentate ligand. As illustrated in Fig. 2, The Zn^II^ cations are connected by OH-bdc and bbie ligands to form a layer parallel to the *ab* plane. There exist hydrogen-binding interactions among adjacent layers. The layers are further connected by these hydrogen bonds to a 3D supramolecular architecture.

### Experimental

The ligand bbie was synthesized according to the literature Liu *et al.*, (2007), but 2-phenylimidazole was replaced by 2-ethylimidazole. A mixture of ZnCO_3_ (0.050 g, 0.40 mmol), OH—H^2^bdc (0.043 g, 0.40 mmol), bbie (0.099 g, 0.40 mmol), and water (8 ml) was sealed in a Teflon reactor (18 ml) and heated at 140 °C for 3 days. After the mixture had been cooled to room temperature at 10 °C.h^-1^, colorless crystals of (I) were obtained. Yield: 43%.

### Refinement

Disorderd bbie ligand was refined using C17 and C18 atoms split over two sites, with a total occupancy of 1. A l l H-atoms bound to carbon were refined using a riding model with d(C—H) = 0.93 Å, *U*_iso_ = 1.2*U*_eq_(C) for aromatic and 0.97 Å, *U*_iso_ = 1.5*U*_eq_(C) for CH_2_ atoms. Hydroxyl H atoms were refined using a riding model with d(O—H) = 0.82 Å, *U*_iso_ =1.5*U*_eq_(O).

### Crystal data

**Table:**

[Zn(C_8_H_4_O_5_)(C_14_H_22_N_4_)]	*F*(000) = 1024
*M**_r_* = 491.84	*D*_x_ = 1.436 Mg m^−^^3^
Monoclinic, *P*2_1_/*n*	Mo *K*α radiation, λ = 0.71069 Å
Hall symbol: -P 2yn	Cell parameters from 3988 reflections
*a* = 13.5120 (12) Å	θ = 1.9–28.3°
*b* = 12.765 (1) Å	µ = 1.12 mm^−^^1^
*c* = 13.5910 (12) Å	*T* = 293 K
β = 103.933 (2)°	Block, colorless
*V* = 2275.2 (3) Å^3^	0.28 × 0.24 × 0.21 mm
*Z* = 4	

### Data collection

**Table:**

Bruker SMART APEX CCD area-detector diffractometer	5467 independent reflections
Radiation source: fine-focus sealed tube	3988 reflections with *I* > 2σ(*I*)
graphite	*R*_int_ = 0.034
ω scans	θ_max_ = 28.3°, θ_min_ = 1.9°
Absorption correction: multi-scan (*SADABS*; Sheldrick, 1996)	*h* = −13→17
*T*_min_ = 0.73, *T*_max_ = 0.79	*k* = −14→17
13890 measured reflections	*l* = −18→18

### Refinement

**Table:**

Refinement on *F*^2^	Primary atom site location: structure-invariant direct methods
Least-squares matrix: full	Secondary atom site location: difference Fourier map
*R*[*F*^2^ > 2σ(*F*^2^)] = 0.036	Hydrogen site location: inferred from neighbouring sites
*wR*(*F*^2^) = 0.087	H-atom parameters constrained
*S* = 1.03	*w* = 1/[σ^2^(*F*_o_^2^) + (0.0374*P*)^2^ + 0.1596*P*] where *P* = (*F*_o_^2^ + 2*F*_c_^2^)/3
5467 reflections	(Δ/σ)_max_ = 0.001
309 parameters	Δρ_max_ = 0.32 e Å^−^^3^
0 restraints	Δρ_min_ = −0.29 e Å^−^^3^

### Special details

**Table:**

Geometry. All e.s.d.'s (except the e.s.d. in the dihedral angle between two l.s. planes) are estimated using the full covariance matrix. The cell e.s.d.'s are taken into account individually in the estimation of e.s.d.'s in distances, angles and torsion angles; correlations between e.s.d.'s in cell parameters are only used when they are defined by crystal symmetry. An approximate (isotropic) treatment of cell e.s.d.'s is used for estimating e.s.d.'s involving l.s. planes.
Refinement. Refinement of *F*^2^ against ALL reflections. The weighted *R*-factor *wR* and goodness of fit *S* are based on *F*^2^, conventional *R*-factors *R* are based on *F*, with *F* set to zero for negative *F*^2^. The threshold expression of *F*^2^ > σ(*F*^2^) is used only for calculating *R*-factors(gt) *etc*. and is not relevant to the choice of reflections for refinement. *R*-factors based on *F*^2^ are statistically about twice as large as those based on *F*, and *R*- factors based on ALL data will be even larger.

### Fractional atomic coordinates and isotropic or equivalent isotropic displacement parameters (Å^2^)

**Table:**

	*x*	*y*	*z*	*U*_iso_*/*U*_eq_	Occ. (<1)
Zn1	0.582734 (18)	0.485563 (18)	0.336590 (17)	0.03285 (9)	
C1	0.35185 (15)	0.26820 (15)	0.21865 (15)	0.0328 (4)	
C2	0.30834 (15)	0.23700 (16)	0.11970 (15)	0.0354 (5)	
H2	0.3183	0.2771	0.0658	0.042*	
C3	0.24997 (15)	0.14625 (16)	0.10089 (15)	0.0339 (5)	
C4	0.23245 (15)	0.08795 (16)	0.18177 (15)	0.0338 (5)	
H4	0.1928	0.0276	0.1695	0.041*	
C5	0.27431 (15)	0.12020 (16)	0.28070 (15)	0.0349 (5)	
C6	0.33484 (16)	0.20925 (16)	0.29951 (15)	0.0358 (5)	
H6	0.3640	0.2296	0.3659	0.043*	
C7	0.41726 (16)	0.36460 (16)	0.23657 (16)	0.0349 (5)	
C8	0.20549 (16)	0.11336 (17)	−0.00696 (16)	0.0368 (5)	
C9	0.48306 (15)	0.70092 (16)	0.27233 (15)	0.0355 (5)	
C10	0.51820 (17)	0.67354 (18)	0.43504 (16)	0.0414 (5)	
H10	0.5403	0.6413	0.4978	0.050*	
C11	0.47890 (18)	0.77010 (18)	0.41969 (18)	0.0466 (6)	
H11	0.4683	0.8162	0.4691	0.056*	
C12	0.46889 (18)	0.68667 (19)	0.16100 (16)	0.0464 (6)	
H12A	0.4015	0.7119	0.1275	0.056*	
H12B	0.4707	0.6122	0.1472	0.056*	
C13	0.5467 (2)	0.7414 (2)	0.1135 (2)	0.0665 (8)	
H13A	0.5387	0.7173	0.0451	0.100*	
H13B	0.6144	0.7255	0.1521	0.100*	
H13C	0.5360	0.8158	0.1134	0.100*	
C14	0.7862 (2)	1.0000 (2)	0.4101 (2)	0.0739 (9)	
H14	0.7847	0.9867	0.4770	0.089*	
C15	0.8584 (2)	0.9700 (2)	0.36442 (19)	0.0632 (7)	
H15	0.9166	0.9323	0.3950	0.076*	
C16	0.74705 (18)	1.05462 (18)	0.25407 (18)	0.0443 (5)	
C17	0.6885 (7)	1.1012 (7)	0.1514 (6)	0.062 (2)	0.688 (10)
H17A	0.7064	1.0627	0.0966	0.074*	0.688 (10)
H17B	0.6158	1.0932	0.1447	0.074*	0.688 (10)
C18	0.7127 (4)	1.2139 (4)	0.1428 (5)	0.097 (2)	0.688 (10)
H18A	0.6750	1.2404	0.0786	0.146*	0.688 (10)
H18B	0.7844	1.2218	0.1479	0.146*	0.688 (10)
H18C	0.6943	1.2523	0.1965	0.146*	0.688 (10)
C18'	0.6330 (13)	1.0952 (16)	0.0904 (14)	0.156 (10)	0.312 (10)
H18D	0.6135	1.1524	0.0439	0.234*	0.312 (10)
H18E	0.5741	1.0694	0.1105	0.234*	0.312 (10)
H18F	0.6617	1.0400	0.0580	0.234*	0.312 (10)
C17'	0.7098 (17)	1.132 (2)	0.1811 (16)	0.099 (10)	0.312 (10)
H17C	0.7669	1.1615	0.1592	0.119*	0.312 (10)
H17D	0.6797	1.1873	0.2129	0.119*	0.312 (10)
C19	0.41398 (18)	0.88519 (17)	0.2671 (2)	0.0536 (6)	
H19A	0.3650	0.9129	0.3021	0.064*	
H19B	0.3777	0.8693	0.1981	0.064*	
C20	0.49432 (19)	0.96907 (18)	0.26511 (19)	0.0514 (6)	
H20A	0.5474	0.9382	0.2374	0.062*	
H20B	0.4627	1.0245	0.2195	0.062*	
C21	0.5432 (2)	1.01699 (19)	0.3666 (2)	0.0540 (7)	
H21A	0.5752	0.9619	0.4125	0.065*	
H21B	0.4905	1.0484	0.3945	0.065*	
C22	0.6228 (2)	1.1000 (2)	0.3613 (2)	0.0618 (7)	
H22A	0.5939	1.1500	0.3084	0.074*	
H22B	0.6408	1.1377	0.4252	0.074*	
N1	0.52069 (13)	0.62938 (13)	0.34288 (12)	0.0348 (4)	
N2	0.45730 (14)	0.78804 (13)	0.31714 (14)	0.0407 (4)	
N3	0.71500 (15)	1.05383 (16)	0.34053 (15)	0.0490 (5)	
N4	0.83402 (14)	1.00315 (13)	0.26584 (13)	0.0379 (4)	
O1	0.48724 (12)	0.36689 (12)	0.31780 (11)	0.0484 (4)	
O2	0.40181 (12)	0.43621 (12)	0.17278 (11)	0.0460 (4)	
O3	0.21788 (14)	0.16868 (13)	−0.07723 (11)	0.0565 (5)	
O4	0.15489 (12)	0.02734 (11)	−0.01974 (10)	0.0400 (4)	
O5	0.26148 (12)	0.06464 (12)	0.36316 (10)	0.0482 (4)	
H5	0.2121	0.0257	0.3459	0.072*	

### Atomic displacement parameters (Å^2^)

**Table:**

	*U*^11^	*U*^22^	*U*^33^	*U*^12^	*U*^13^	*U*^23^
Zn1	0.03576 (15)	0.03453 (14)	0.02655 (13)	−0.00202 (10)	0.00417 (10)	0.00010 (10)
C1	0.0291 (11)	0.0328 (11)	0.0340 (11)	−0.0004 (8)	0.0028 (9)	−0.0028 (9)
C2	0.0374 (12)	0.0369 (11)	0.0307 (10)	−0.0007 (9)	0.0057 (9)	0.0014 (9)
C3	0.0328 (11)	0.0366 (11)	0.0297 (10)	0.0016 (9)	0.0023 (9)	−0.0023 (9)
C4	0.0302 (11)	0.0338 (11)	0.0350 (11)	−0.0027 (9)	0.0032 (9)	−0.0004 (9)
C5	0.0334 (11)	0.0396 (12)	0.0310 (11)	0.0013 (9)	0.0062 (9)	0.0041 (9)
C6	0.0372 (12)	0.0393 (12)	0.0274 (10)	−0.0012 (9)	0.0006 (9)	−0.0048 (9)
C7	0.0341 (12)	0.0373 (11)	0.0342 (11)	−0.0003 (9)	0.0095 (9)	−0.0065 (9)
C8	0.0360 (12)	0.0382 (12)	0.0326 (11)	0.0019 (9)	0.0014 (9)	−0.0008 (9)
C9	0.0331 (11)	0.0359 (11)	0.0365 (11)	−0.0023 (9)	0.0065 (9)	0.0013 (9)
C10	0.0458 (13)	0.0450 (13)	0.0352 (12)	−0.0027 (10)	0.0130 (10)	−0.0027 (10)
C11	0.0515 (15)	0.0464 (14)	0.0465 (14)	−0.0030 (11)	0.0211 (12)	−0.0109 (11)
C12	0.0518 (14)	0.0470 (14)	0.0370 (12)	0.0041 (11)	0.0042 (11)	0.0047 (10)
C13	0.089 (2)	0.0669 (18)	0.0512 (15)	−0.0035 (16)	0.0321 (15)	0.0076 (13)
C14	0.076 (2)	0.112 (3)	0.0406 (15)	0.0171 (18)	0.0274 (15)	0.0192 (15)
C15	0.0628 (18)	0.088 (2)	0.0404 (14)	0.0194 (15)	0.0163 (13)	0.0225 (14)
C16	0.0472 (14)	0.0423 (13)	0.0471 (13)	0.0010 (11)	0.0186 (11)	0.0030 (11)
C17	0.060 (4)	0.074 (4)	0.044 (4)	0.027 (3)	−0.001 (3)	0.006 (3)
C18	0.090 (4)	0.082 (4)	0.112 (5)	0.013 (3)	0.011 (3)	0.059 (3)
C18'	0.092 (12)	0.26 (2)	0.110 (13)	0.064 (14)	0.018 (10)	0.090 (14)
C17'	0.066 (12)	0.19 (3)	0.055 (12)	0.051 (13)	0.036 (10)	0.035 (12)
C19	0.0472 (15)	0.0385 (13)	0.0748 (18)	0.0058 (11)	0.0140 (13)	0.0050 (12)
C20	0.0549 (16)	0.0382 (13)	0.0642 (17)	0.0024 (11)	0.0202 (13)	0.0052 (12)
C21	0.0518 (15)	0.0517 (15)	0.0673 (17)	−0.0062 (12)	0.0312 (14)	−0.0104 (13)
C22	0.0590 (17)	0.0528 (16)	0.086 (2)	−0.0054 (13)	0.0416 (15)	−0.0192 (14)
N1	0.0374 (10)	0.0359 (10)	0.0310 (9)	−0.0003 (8)	0.0081 (8)	−0.0001 (8)
N2	0.0395 (11)	0.0332 (10)	0.0507 (11)	0.0010 (8)	0.0133 (9)	−0.0009 (8)
N3	0.0498 (12)	0.0519 (12)	0.0509 (12)	−0.0039 (10)	0.0231 (10)	−0.0098 (10)
N4	0.0409 (11)	0.0412 (10)	0.0325 (9)	0.0015 (8)	0.0104 (8)	0.0013 (8)
O1	0.0477 (10)	0.0477 (9)	0.0418 (9)	−0.0153 (8)	−0.0048 (7)	0.0006 (7)
O2	0.0533 (10)	0.0365 (9)	0.0454 (9)	−0.0049 (7)	0.0066 (8)	0.0031 (7)
O3	0.0758 (12)	0.0585 (11)	0.0317 (8)	−0.0190 (9)	0.0063 (8)	0.0016 (8)
O4	0.0453 (9)	0.0381 (8)	0.0312 (8)	−0.0052 (7)	−0.0013 (7)	−0.0039 (6)
O5	0.0563 (10)	0.0549 (10)	0.0313 (8)	−0.0147 (8)	0.0066 (7)	0.0049 (7)

### Geometric parameters (Å, °)

**Table:**

Zn1—O1	1.9662 (15)	C14—H14	0.9300
Zn1—O4^i^	1.9682 (14)	C15—N4	1.367 (3)
Zn1—N4^ii^	2.0020 (18)	C15—H15	0.9300
Zn1—N1	2.0286 (17)	C16—N4	1.322 (3)
C1—C2	1.390 (3)	C16—N3	1.347 (3)
C1—C6	1.396 (3)	C16—C17'	1.40 (3)
C1—C7	1.500 (3)	C16—C17	1.547 (9)
C2—C3	1.390 (3)	C17—C18	1.486 (12)
C2—H2	0.9300	C17—H17A	0.9700
C3—C4	1.394 (3)	C17—H17B	0.9700
C3—C8	1.504 (3)	C18—H18A	0.9600
C4—C5	1.390 (3)	C18—H18B	0.9600
C4—H4	0.9300	C18—H18C	0.9600
C5—O5	1.372 (2)	C18'—C17'	1.48 (3)
C5—C6	1.388 (3)	C18'—H18D	0.9600
C6—H6	0.9300	C18'—H18E	0.9600
C7—O2	1.243 (2)	C18'—H18F	0.9600
C7—O1	1.269 (2)	C17'—H17C	0.9700
C8—O3	1.231 (3)	C17'—H17D	0.9700
C8—O4	1.283 (2)	C19—N2	1.467 (3)
C9—N1	1.333 (2)	C19—C20	1.530 (3)
C9—N2	1.353 (3)	C19—H19A	0.9700
C9—C12	1.490 (3)	C19—H19B	0.9700
C10—C11	1.338 (3)	C20—C21	1.508 (3)
C10—N1	1.381 (3)	C20—H20A	0.9700
C10—H10	0.9300	C20—H20B	0.9700
C11—N2	1.373 (3)	C21—C22	1.524 (3)
C11—H11	0.9300	C21—H21A	0.9700
C12—C13	1.530 (3)	C21—H21B	0.9700
C12—H12A	0.9700	C22—N3	1.466 (3)
C12—H12B	0.9700	C22—H22A	0.9700
C13—H13A	0.9600	C22—H22B	0.9700
C13—H13B	0.9600	N4—Zn1^iii^	2.0020 (18)
C13—H13C	0.9600	O4—Zn1^iv^	1.9682 (14)
C14—C15	1.333 (4)	O5—H5	0.8200
C14—N3	1.362 (3)		
			
O1—Zn1—O4^i^	102.54 (6)	C18—C17—H17A	109.3
O1—Zn1—N4^ii^	117.20 (7)	C16—C17—H17A	109.3
O4^i^—Zn1—N4^ii^	118.18 (7)	C18—C17—H17B	109.3
O1—Zn1—N1	115.96 (7)	C16—C17—H17B	109.3
O4^i^—Zn1—N1	98.33 (6)	H17A—C17—H17B	108.0
N4^ii^—Zn1—N1	103.59 (7)	C17—C18—H18A	109.5
C2—C1—C6	119.72 (18)	C17—C18—H18B	109.5
C2—C1—C7	119.17 (18)	H18A—C18—H18B	109.5
C6—C1—C7	121.11 (17)	C17—C18—H18C	109.5
C3—C2—C1	120.38 (19)	H18A—C18—H18C	109.5
C3—C2—H2	119.8	H18B—C18—H18C	109.5
C1—C2—H2	119.8	C17'—C18'—H18D	109.5
C2—C3—C4	119.76 (18)	C17'—C18'—H18E	109.5
C2—C3—C8	119.19 (19)	H18D—C18'—H18E	109.5
C4—C3—C8	121.04 (18)	C17'—C18'—H18F	109.5
C5—C4—C3	119.87 (19)	H18D—C18'—H18F	109.5
C5—C4—H4	120.1	H18E—C18'—H18F	109.5
C3—C4—H4	120.1	C16—C17'—C18'	115 (2)
O5—C5—C6	117.24 (17)	C16—C17'—H17C	108.4
O5—C5—C4	122.36 (19)	C18'—C17'—H17C	108.4
C6—C5—C4	120.36 (19)	C16—C17'—H17D	108.4
C5—C6—C1	119.87 (18)	C18'—C17'—H17D	108.4
C5—C6—H6	120.1	H17C—C17'—H17D	107.5
C1—C6—H6	120.1	N2—C19—C20	113.23 (19)
O2—C7—O1	123.70 (19)	N2—C19—H19A	108.9
O2—C7—C1	119.78 (18)	C20—C19—H19A	108.9
O1—C7—C1	116.52 (18)	N2—C19—H19B	108.9
O3—C8—O4	123.61 (19)	C20—C19—H19B	108.9
O3—C8—C3	119.93 (19)	H19A—C19—H19B	107.7
O4—C8—C3	116.46 (18)	C21—C20—C19	115.1 (2)
N1—C9—N2	109.52 (18)	C21—C20—H20A	108.5
N1—C9—C12	125.47 (19)	C19—C20—H20A	108.5
N2—C9—C12	124.99 (19)	C21—C20—H20B	108.5
C11—C10—N1	109.3 (2)	C19—C20—H20B	108.5
C11—C10—H10	125.4	H20A—C20—H20B	107.5
N1—C10—H10	125.4	C20—C21—C22	113.4 (2)
C10—C11—N2	106.9 (2)	C20—C21—H21A	108.9
C10—C11—H11	126.5	C22—C21—H21A	108.9
N2—C11—H11	126.5	C20—C21—H21B	108.9
C9—C12—C13	116.1 (2)	C22—C21—H21B	108.9
C9—C12—H12A	108.3	H21A—C21—H21B	107.7
C13—C12—H12A	108.3	N3—C22—C21	111.8 (2)
C9—C12—H12B	108.3	N3—C22—H22A	109.3
C13—C12—H12B	108.3	C21—C22—H22A	109.3
H12A—C12—H12B	107.4	N3—C22—H22B	109.3
C12—C13—H13A	109.5	C21—C22—H22B	109.3
C12—C13—H13B	109.5	H22A—C22—H22B	107.9
H13A—C13—H13B	109.5	C9—N1—C10	106.52 (17)
C12—C13—H13C	109.5	C9—N1—Zn1	132.81 (14)
H13A—C13—H13C	109.5	C10—N1—Zn1	120.54 (14)
H13B—C13—H13C	109.5	C9—N2—C11	107.76 (18)
C15—C14—N3	107.1 (2)	C9—N2—C19	127.13 (19)
C15—C14—H14	126.4	C11—N2—C19	125.11 (19)
N3—C14—H14	126.4	C16—N3—C14	106.8 (2)
C14—C15—N4	109.7 (2)	C16—N3—C22	129.2 (2)
C14—C15—H15	125.1	C14—N3—C22	124.1 (2)
N4—C15—H15	125.1	C16—N4—C15	105.6 (2)
N4—C16—N3	110.7 (2)	C16—N4—Zn1^iii^	127.12 (15)
N4—C16—C17'	126.3 (9)	C15—N4—Zn1^iii^	127.13 (17)
N3—C16—C17'	119.1 (9)	C7—O1—Zn1	117.32 (14)
N4—C16—C17	123.1 (4)	C8—O4—Zn1^iv^	109.05 (13)
N3—C16—C17	126.0 (4)	C5—O5—H5	109.5
C18—C17—C16	111.6 (6)		
			
C6—C1—C2—C3	−1.4 (3)	O1—Zn1—N1—C9	−91.05 (19)
C7—C1—C2—C3	178.03 (18)	O4^i^—Zn1—N1—C9	160.56 (18)
C1—C2—C3—C4	1.9 (3)	N4^ii^—Zn1—N1—C9	38.8 (2)
C1—C2—C3—C8	−178.86 (19)	O1—Zn1—N1—C10	93.79 (16)
C2—C3—C4—C5	−0.7 (3)	O4^i^—Zn1—N1—C10	−14.60 (17)
C8—C3—C4—C5	−179.88 (18)	N4^ii^—Zn1—N1—C10	−136.36 (16)
C3—C4—C5—O5	−178.50 (19)	N1—C9—N2—C11	−0.5 (2)
C3—C4—C5—C6	−1.0 (3)	C12—C9—N2—C11	178.1 (2)
O5—C5—C6—C1	179.13 (18)	N1—C9—N2—C19	179.53 (19)
C4—C5—C6—C1	1.5 (3)	C12—C9—N2—C19	−1.9 (3)
C2—C1—C6—C5	−0.3 (3)	C10—C11—N2—C9	0.7 (2)
C7—C1—C6—C5	−179.75 (19)	C10—C11—N2—C19	−179.3 (2)
C2—C1—C7—O2	28.3 (3)	C20—C19—N2—C9	−95.4 (3)
C6—C1—C7—O2	−152.2 (2)	C20—C19—N2—C11	84.6 (3)
C2—C1—C7—O1	−151.0 (2)	N4—C16—N3—C14	0.6 (3)
C6—C1—C7—O1	28.4 (3)	C17'—C16—N3—C14	−158.6 (11)
C2—C3—C8—O3	−2.7 (3)	C17—C16—N3—C14	176.4 (5)
C4—C3—C8—O3	176.5 (2)	N4—C16—N3—C22	−179.4 (2)
C2—C3—C8—O4	177.98 (19)	C17'—C16—N3—C22	21.4 (12)
C4—C3—C8—O4	−2.8 (3)	C17—C16—N3—C22	−3.7 (6)
N1—C10—C11—N2	−0.7 (3)	C15—C14—N3—C16	0.0 (3)
N1—C9—C12—C13	−104.2 (3)	C15—C14—N3—C22	180.0 (2)
N2—C9—C12—C13	77.4 (3)	C21—C22—N3—C16	105.4 (3)
N3—C14—C15—N4	−0.6 (4)	C21—C22—N3—C14	−74.7 (3)
N4—C16—C17—C18	−96.2 (6)	N3—C16—N4—C15	−1.0 (3)
N3—C16—C17—C18	88.6 (7)	C17'—C16—N4—C15	156.4 (12)
C17'—C16—C17—C18	10 (3)	C17—C16—N4—C15	−176.8 (5)
N4—C16—C17'—C18'	99.1 (17)	N3—C16—N4—Zn1^iii^	−177.31 (14)
N3—C16—C17'—C18'	−105.3 (15)	C17'—C16—N4—Zn1^iii^	−19.9 (12)
C17—C16—C17'—C18'	9.4 (19)	C17—C16—N4—Zn1^iii^	6.8 (5)
N2—C19—C20—C21	−69.9 (3)	C14—C15—N4—C16	0.9 (3)
C19—C20—C21—C22	179.9 (2)	C14—C15—N4—Zn1^iii^	177.28 (19)
C20—C21—C22—N3	−70.7 (3)	O2—C7—O1—Zn1	−5.3 (3)
N2—C9—N1—C10	0.1 (2)	C1—C7—O1—Zn1	173.94 (13)
C12—C9—N1—C10	−178.5 (2)	O4^i^—Zn1—O1—C7	168.10 (15)
N2—C9—N1—Zn1	−175.60 (14)	N4^ii^—Zn1—O1—C7	−60.73 (17)
C12—C9—N1—Zn1	5.8 (3)	N1—Zn1—O1—C7	62.23 (17)
C11—C10—N1—C9	0.4 (2)	O3—C8—O4—Zn1^iv^	−10.2 (3)
C11—C10—N1—Zn1	176.70 (15)	C3—C8—O4—Zn1^iv^	169.17 (14)

Symmetry codes: (i) *x*+1/2, −*y*+1/2, *z*+1/2; (ii) −*x*+3/2, *y*−1/2, −*z*+1/2; (iii) −*x*+3/2, *y*+1/2, −*z*+1/2; (iv) *x*−1/2, −*y*+1/2, *z*−1/2.

### Hydrogen-bond geometry (Å, °)

**Table:**

*D*—H···*A*	*D*—H	H···*A*	*D*···*A*	*D*—H···*A*
C10—H10···O1^v^	0.93	2.62	3.417 (3)	144
C14—H14···O5^v^	0.93	2.49	3.398 (3)	166
O5—H5···O2^vi^	0.82	1.88	2.697 (2)	171

Symmetry codes: (v) −*x*+1, −*y*+1, −*z*+1; (vi) −*x*+1/2, *y*−1/2, −*z*+1/2.
